# Pregnancy-related issues in women with multiple sclerosis: an evidence-based review with practical recommendations

**DOI:** 10.1080/21556660.2020.1721507

**Published:** 2020-02-06

**Authors:** Beatriz Canibaño, Dirk Deleu, Boulenouar Mesraoua, Gayane Melikyan, Faiza Ibrahim, Yolande Hanssens

**Affiliations:** a Department of Neurology, Neuroscience Institute, Hamad Medical Corporation, Doha, Qatar; b Clinical Services Unit, Pharmacy, Hamad Medical Corporation, Doha, Qatar

**Keywords:** Multiple sclerosis, pregnancy, breastfeeding, delivery, newborn, disease-modifying therapy, postpartum

## Abstract

**Objective:**

To review the current evidence regarding pregnancy-related issues in multiple sclerosis (MS) and to provide recommendations specific for each of them.

**Research design and methods:**

A systematic review was performed based on a comprehensive literature search.

**Results:**

MS has no effect on fertility, pregnancy or fetal outcomes, and pregnancies do not affect the long-term disease course and accumulation of disability. There is a potential risk for relapse after use of gonadotropin-releasing hormone agonists during assisted reproduction techniques. At short-term, pregnancy leads to a reduction of relapses during the third trimester, followed by an increased risk of relapses during the first three months postpartum. Pregnancies in MS are not *per se* high risk pregnancies, and MS does not influence the mode of delivery or anesthesia unless in the presence of significant disability. MRI is not contraindicated during pregnancy; however, gadolinium contrast media should be avoided whenever possible. It is safe to use pulse dose methylprednisolone infusions to manage acute disabling relapses during pregnancy and breastfeeding. However, its use during the first trimester of pregnancy is still controversial. Women with MS should be encouraged to breastfeed with a possible favorable effect of exclusive breastfeeding. Disease-modifying drugs can be classified according to their potential for pregnancy-associated risk and impact on fetal outcome. Interferon beta (IFNβ) and glatiramer acetate (GA) may be continued until pregnancy is confirmed and, after consideration of the individual risk-benefit if continued, during pregnancy. The benefit of continuing natalizumab during the entire pregnancy may outweigh the risk of recurring disease activity, particularly in women with highly active MS. GA and IFNβ are considered safe during breastfeeding. The use of natalizumab during pregnancy or lactation requires monitoring of the newborn.

**Conclusions:**

This review provides current evidence and recommendations for counseling and management of women with MS preconception, during pregnancy and postpartum.

## Introduction

Multiple sclerosis (MS) is a chronic autoimmune condition affecting the central nervous system (CNS). Pathologically, the disease is characterized by diffuse and focal areas of inflammation, demyelination, gliosis and axonal injury in the brain and spinal cord. Relapsing–remitting MS (RRMS) is the most common form (85–90% of patients at disease onset)^1^^,^^2^. Relapses being the clinical expression of the acute inflammatory process, and progression being related to chronic diffuse axonal and neuronal degeneration^1^. MS is the leading medical cause of neurological disability typically starting in the third and fourth decades of life and preferentially affects young females (female/male ratio ∼3) potentially impacting a woman’s reproductive years^2–4^. Lack of education, especially with regard to treatment options during pregnancy, is a significant concern among women with MS. A recent study in female patients with MS, revealed that 47% of patients felt inadequately informed about their disease-modifying therapy (DMT) use during pregnancy^5^. This highlights the importance of discussing with the patient reproductive and pregnancy related-issues including impact of MS on fertility and fetal development; MS medications before, during and after pregnancy; effect of pregnancy on the MS prognosis in short- and long-term; DMT use and recommended washout periods, monitoring of relapses and disease activity during pregnancy, delivery and anesthetic choice, postpartum disease activity and breastfeeding.

The objective of this review is to assess the current evidence regarding the short-term effects of pregnancy on MS and provide evidence- and experience-based practical guidance for the treatment of MS in women of childbearing age before conception, during pregnancy and lactation.

## Methods

A systematic literature review was performed through a comprehensive search of MEDLINE, PubMed, Embase, Cochrane Database of Systematic Reviews (period 1 January 1995 through 30 June 2019) using a combination of medical subject headings (MeSH) and free-text terms to identify publications of relevant studies on pregnancy, breastfeeding and MS. The following search terms were used either AND or OR depending the search term: “multiple sclerosis”, “pregnancy”, “pregnant”,” gestation”, “breastfeeding”, “lactation”, “guidelines”, “recommendations”, “algorithm”. In addition a search was conducted for individual DMTs used in the management of MS together with the above search terms. Conference abstracts were included and identified *via* Embase or web-searches of relevant conference websites. Additional searches were performed of American Academy of Neurology (AAN) and European Committee for Treatment and Research in Multiple Sclerosis (ECTRIMS) abstracts from 2012 thru 2019 using identical search strategies on their respective websites. Articles and abstracts retrieved needed to be original reports, and of relevance to the scope of this manuscript. Full-text versions of articles were examined. No language restrictions were imposed on the retrieved articles.

The following outcomes were assessed: (1) fatherhood in MS; (2) the effect of pregnancy on MS relapse rate and *vice versa*; (3) pregnancy complications, delivery and anesthetic mode; (4) the relative risk of prematurity and low birth weight; (5) the prevalence of malformations; (6) the effect of breastfeeding on MS; (7) the effect of MRI and corticosteroids during pregnancy and postpartum period; (8) the effect of different DMTs on pregnancy and breastfeeding.

This study was granted approval by the Medical Research and Ethics Committee of our institution.

## Definition of disease activity

There are no consensus criteria for defining the activity of MS. The European Medicines Agency (EMA) recommends consideration of progression of the clinical burden of disease (number of relapses, worsening disability and increasing radiologic burden) in defining MS activity^6^. We have defined mild disease (moderate disease would show features of both) and highly active disease that follow the criteria used within the EMA’s evaluation of fingolimod and natalizumab^7^^,^^8^. These criteria provide a useful and practical guide to assessing disease activity, but do not take into consideration individual patient or prognostic factors, such as older age at presentation, incomplete recovery from relapses, the presence of motor relapses at presentation, rapid progression of disability (e.g. increase of at least one Expanded Disability Status Scale (EDSS) point in one year), or presentation with spinal, or cerebellar or brainstem lesions.

Mild RRMS is defined: (1) in treatment-naïve patients, as ≤1 disabling relapse in the previous year, or no gadolinium-enhancing lesions, and no significant increase in T2 lesion load compared with a recent MRI; (2) in patients treated with at least one DMT, decreased relapse rate and no ongoing severe relapses, no relapses in the past year or no gadolinium-enhancing lesions and ≤8 T2 lesions.

Highly active RRMS should have at least one of the following: (1) in a treatment-naïve patient, ≥2 relapses in the previous year with ≥1 gadolinium-enhancing lesions on brain MRI or a significant increase in T2 lesion load as compared to a previous recent MRI; (2) suboptimal treatment response to an adequate course of ≥1 DMT(s) and presenting with ≥1 relapse in the previous year while on therapy and having ≥9 T2 lesions or ≥1 gadolinium-enhancing lesion.

## Fatherhood in patients with MS

Men with MS may present fertility impairment and sexual dysfunction, while semen itself may be affected by the DMT. There are limited data on the potential course and outcome of pregnancies fathered by men suffering from MS while on DMTs. In a large population-based study in which either interferon-β (IFNβ) or glatiramer acetate (GA) were used in 202 pregnancies fathered by 141 men with MS, no difference in mean birth weight and gestational age was found compared with fathers without MS^9^. Furthermore, paternal MS and MS-related clinical factors were not significantly associated with birth outcomes. Data are still missing on the impact of the other DMTs used for MS on male fertility.

The potential foetotoxic effect of the different DMTs in paternal MS will be discussed in the corresponding paragraphs.

*Recommendation*: Current evidence does not suggest that paternal MS affects fertility or birth outcomes.

## Genetic risks for transmitting MS to offspring

MS is essentially not an inherited disorder. The etiopathogenesis of MS is likely based on the intricate interaction between genetic factors and environmental triggers. The strongest genetic risk factor is linked to HLA (HLA DRB1*15), while smoking, lack of vitamin D, and infection with Epstein-Barr virus are well-known environmental factors^10^^,^^11^. The risk for developing MS in the general population is low (0.1–0.3%)^2^. When having a first-degree relative with MS the risk increases to 2–4%. In conjugal MS, this risk increases further to 6–12%^12^^,^^13^. About 15% of all MS patients have a close or distant relative with MS but this increase might be subject to geographical area and parental consanguinity^14^.

*Recommendation*: MS is not an inherited disease, but there is genetic risk that may be inherited.

## Fetal development

Compared with healthy women, MS does not appear to have an impact on fetal development. Some studies indicate that women with MS may have higher risk for delivering neonates with lower birth weight compared with healthy women^15–17^. Thus far no long-term adverse pediatric outcomes have been reported^18^^,^^19^.

While the amount of data available on DMT safety in women who become pregnant is increasing, few DMTs are currently considered entirely safe for use during pregnancy. The decision to continue DMT depends on disease severity and the DMT in question. The potential impact on fetal development of each one of the DMTs will be discussed in the corresponding section.

*Recommendation*: MS has no impact on fetal development.

## Fertility and assisted reproduction techniques (ART)

The effect of MS on fertility is still a matter of debate. Blood hormone abnormalities and sexual dysfunction may affect fertility which have been observed in female patients with MS^15^^,^^20^. Despite this, population-based studies seem to indicate that spontaneous pregnancies and time to pregnancy do not appear to be any different from the general population^21^^,^^22^. The use of certain immunosuppressant agents such as mitoxantrone and cyclophosphamide, is associated with reduced fertility and reproductive toxicity. There is ample evidence from clinical observations^23–26^ that ART – independently of the type of hormonal treatment used – increases the relapse risk, the number of new or enlarging T2 lesions and gadolinium-enhancing lesions, particularly in the first 3 months after failing to conceive^15^^,^^25^. Gonadotropin-releasing hormone (GnRH) agonists are known to stimulate immune cell proliferation; cytokine, chemokine and endothelial growth factor production; as well as estrogen levels. The use of GnRH antagonists seems to carry less of a risk but this still needs to be confirmed in proper prospective studies^27^. There is some evidence that maintaining the patient on GA or IFNβ during the procedure until conception, may help preventing relapse risk^28^.

*Recommendation*: Use of GnRH agonists during ART carries a potential risk for having a relapse.

## Preconception period in women with MS

Pregnancy is still a major concern for the majority of woman diagnosed with MS. Recently, an Italian study investigated childlessness in female MS patients (*N* = 303) and age-matched controls (*N* = 500) once they reached the end of their reproductive period (>43 years of age)^29^. Sixty-seven MS women (22%) were childless compared with 66 controls (13%). The most cited reason for childlessness was lack of stable relationship followed by no childbearing desires. Female MS patients and age-matched controls had their first child at the same age (27 years of age) and the study did not suggest impaired fertility in women with MS.

At this stage, counseling is essential and should be provided to all women with MS and their partners, at or soon after the diagnosis, to inform them on MS and pregnancy, and to anticipate any concerns.

Pregnancy requires careful planning, taking into account the disease activity and severity, and potential impact of DMTs on the pregnancy and fetus. The counseling should also consider the psychological and cognitive status of the patient. Women of childbearing potential should not defer DMT because of their wish to become pregnant. In this respect, and similar to other cell-mediated autoimmune diseases timing might be crucial as there is no evidence that any form of contraception affects the clinical course of MS^30^. Particularly, oral contraceptives with high dose estrogens may have anti-inflammatory effects and when used in combination with IFNβ they are effective in reducing the rate of development of new T2 lesions on MRI^31^.

MS-specific preconception considerations include avoidance of alcohol and smoking, and assure good sleep hygiene as well as a balanced diet^22^^,^^32^. The role of maternal vitamin D deficiency during pregnancy has been well-established. The association of maternal 25(OH) vitamin D levels <12 ng/mL during early pregnancy result in nearly twofold higher risk of MS in the offspring, compared with women who did not have deficient vitamin D levels^22^^,^^33^^,^^34^. Therefore, women with MS are advised to take vitamin D at the average dosage of 1,000–2,000 units/day prior to conception and to ensure levels are between 30 and 40 ng/mL^35^. In addition, and as standard recommendation for all pregnancies, periconceptional folic acid supplementation (0.4–0.8 g oral per daily) decreases the occurrence and recurrence of neural tube defects. Special attention should also be paid to the potential for urinary tract infections.

Inactivated vaccines, e.g. seasonal influenza and tetanus, can be used safely at any stage during pregnancy. Vaccines with bacteria (Bacillus Calmette–Guérin (BCG), typhoid) and those with attenuated viruses (measles mumps-rubella, varicella/zoster and rotavirus) are safe during preconception and in the last trimester of pregnancy^36^. If needed, polysaccharide pneumococcal and hepatitis B vaccination may be used during pregnancy. However live-virus vaccines (e.g. yellow fever) should be avoided in pregnant women with MS.

In women with MS who wish to conceive – including newly diagnosed patients – conception should be delayed until the disease is adequately controlled with a DMT (1–2 years depending on disease activity), unless there is a concern about rapidly declining fecundity due to advanced age^37^.

*Recommendations*: Counseling is essential and should be provided to all women with MS and their partners at or soon after the diagnosis, to inform them on MS and pregnancy, and to anticipate any concerns. Standard pregnancy advice should be provided emphasizing the use of vitamin D. Live-virus vaccine should be avoided during pregnancy. Conception should be delayed until the disease is adequately controlled.

## The effect of MS disease activity and course on pregnancy

Compared with healthy women, MS does not put patients at higher risk for pregnancy complications such as ectopic pregnancy, placental abnormalities, spontaneous abortions, antepartum hemorrhages, preeclampsia, stillbirth, premature birth or congenital malformation^28^^,^^32^^,^^34^.

*Recommendation*: MS does not affect pregnancy outcomes.

## The effect of pregnancy on MS disease activity and course

The impact of pregnancy on the long-term disease course of MS is still a matter of debate. Women who have children after MS onset appear to have much slower progression of EDSS score compared with nulliparous women^38–40^. Term pregnancies prove to have no effect on the time to reach a given disability level, which was predicted only by a progressive course and older age at MS onset^41^. Overall, there is no evidence to conclude that pregnancy or the number of pregnancies has a negative impact on the long-term course or progression to irreversible disability^40–48^. With regard to whether pregnancy has an impact on the risk for developing MS in patients with clinically isolated syndrome, an Australian study revealed that increasing gravidity and parity was associated with a lower risk of developing MS. In addition, there was a 49% reduction in the risk of a first relapse for each child born^48^. Similar findings were observed in a Danish cohort, in which childbirths within five years before clinical onset reduced the risk of MS onset in women for one child with OR = 0.54 and for more than one child with OR = 0.68^49^.

At short-term, the effect of pregnancy on relapses and disability progression was assessed in the PRIMS (Pregnancy in Multiple Sclerosis) study in which 269 DMT-unexposed pregnancies in 254 women with MS were followed-up prospectively during pregnancy and for a period up to 2 years after delivery^50^^,^^51^. The findings revealed that: (1) the annualized relapse rate (ARR) during pregnancy decreased during the third trimester of pregnancy to 0.2 (vs. 0.7 the year before pregnancy); (2) there was an increased ARR (1.2 (vs. 0.7 the year before pregnancy) during the first 3 months postpartum, in which nearly 30% of patients experienced relapses; (3) the overall ARR in the pregnancy year (9 months of pregnancy and 3 months postpartum) was similar to the antepartum rate; (4) the disease activity steadily returned to levels observed preconception^50–52^; (5) no change in disability progression was observed during the study period. Besides poor prognostic profile the following have been identified as predictors for early postpartum relapses: (i) higher ARR in the 2 years preconception, (ii) relapses during pregnancy, (iii) a higher EDSS score at conception and lastly, (iv) lack of prior DMT use 2 years preconception^50^^,^^51^^,^^53–55^. Similarly, women who had received DMDs for at least 8 weeks during pregnancy had lower risk of postpartum relapses compared with women who had not received any DMD during the pregnancy or in the 3 months before conception^56^. These observations would favor – whenever possible and safe – keeping the patient on DMDs until conception.

Postpartum rebound of disease activity is also supported by MRI studies that showed an increase in new or enlarging T2 gadolinium-enhancing lesions postpartum^57^^,^^58^. This suggests that minimizing relapse frequency prior to conception may lead to better early postpartum outcomes. Several studies confirmed the results of PRIMS but observed lower relapse rates than in the PRIMS study in most of the study periods^53^^,^^59^^,^^60^.

*Recommendations*: Counsel women with MS and their partners about the course of the disease prior to conception, during and after pregnancy. Reassure women about the lack of any negative effect of pregnancy on the long-term disease progression.

## Monitoring of disease activity during pregnancy and lactation

A woman with MS should be monitored for any exacerbation of disease activity during pregnancy. If disease reactivation is suspected throughout pregnancy, it is safe to use low-field-strength MRI (1.5 Tesla) without contrast^61^. However, MRI should only be considered if it is absolutely indicated and the findings could have therapeutic consequences. Because of a variety of negative effects on the developing fetus and early childhood, the use of gadolinium is strongly discouraged during any trimester of pregnancy unless the potential clinical benefits to the mother clearly outweigh the risks^62^. All other tests used in patients with MS, including neurophysiological tests and lumbar puncture, are safe during pregnancy but should only be performed if essential for the diagnosis.

With respect to breastfeeding, the excretion of the gadolinium contrast agents into breast milk is <1%, as is its absorption from the infant gut^62^. Hence, the use of gadolinium in the mother is considered safe during lactation without requiring interruption of breastfeeding^63–66^. However if the patient wishes to avoid any gadolinium ingestion at all by the infant, they should “pump and dump” the milk for 24 h after gadolinium contrast exposure.

*Recommendations*: There is no contraindication for MRI at any time during pregnancy, however, gadolinium contrast media should be avoided whenever possible. The use of gadolinium contrast media in the mother is considered safe during lactation.

## Delivery and obstetrical anesthesia

No significant differences are observed between women with MS and the general population with regard to: duration of the mother’s hospital stay after delivery, frequency of assisted vaginal delivery, and frequency of cesarean delivery^67–70^. Although the percentage of women with MS receiving epidural/spinal anesthesia or cesarean section is relatively low, there is no evidence indicating that either of them have an impact on delivery or postpartum relapses or disability progression^50^^,^^51^^,^^71^^,^^72^. Some studies showed a trend for increased cesarean or assisted vaginal delivery with increased MS disability^67^. However, there may be some bias in attitude of the obstetrician toward regional anesthesia and cesarean section particularly in the presence of significant motor disability, and potential fatigue and exhaustion during labor.

*Recommendation*: Pregnancies in women with MS are not per se high risk pregnancies and MS does not influence the mode of delivery or anesthesia unless significant disability is present.

## Management of acute disabling relapse during pregnancy and postpartum period

The safety of corticosteroid during pregnancy varies according to the type of corticosteroid, the trimester of the pregnancy during which the drug is administered and the duration and dosage of the treatment. Relapses during the second and third trimesters can be safely treated with short courses of conventional corticosteroids (commonly 1 g of IV methylprednisolone daily for 3–5 days)^73^. Methylprednisolone is actively metabolized by the placenta to inactive products by 11-β-hydroxysteroid dehydrogenase, allowing less than 10% of the maternal dose to reach the fetus. Dexamethasone and betamethasone cross the placenta with minimal metabolism and are therefore not recommended^73^. Although still controversial, corticosteroids should likely be avoided during the first trimester of pregnancy because of their potential risk for miscarriage, teratogenicity such as craniofacial abnormalities (e.g. orofacial cleft) and low birth weight^73–77^. In view of this, corticosteroids should therefore be restricted for acute disabling relapses that substantially affect activities of daily living^78^^,^^79^.

With regard to breastfeeding following treatment with pulse doses of IV methylprednisolone, it is considered safe to continue breastfeeding provided an interval of 2–4 h after each intravenous dose is applied^80–82^. Corticosteroids appear to be minimally excreted into breast milk. Data from lactating women treated with prednisolone (10–80 mg daily) revealed that the doses of corticosteroids ingested from breast milk would add a negligible 10% to the infant’s endogenous corticosteroids production^73^^,^^82^. Nonetheless, if deemed necessary, breastfeeding can be suspended for 24–48 h after the infusion using a “pump and dump” approach in the interim^73^^,^^77^.

The impact of high-dose methylprednisolone (1 g monthly for 6 months) for the prevention of relapses postpartum remains too poor to draw conclusions^83^.

Intravenous immunoglobulin (IVIG) can be used safely throughout pregnancy and postpartum but it is not effective in the treatment of acute relapses and provides inconsistent results in relapse prevention^84–87^. IVIG has no known adverse effects on the infant^85^. Large controlled clinical trials are required to confirm the role of IVIG in relapse prevention during pregnancy and postpartum^86^^,^^87^.

The use of therapeutic plasma exchange in pregnant MS patients is still anecdotal but could be beneficial as an alternative to corticosteroids in the first trimester of pregnancy and in case of steroid-refractory MS relapse^88^.

*Recommendations*: Acute disabling relapses can be safely treated with IV methylprednisolone and IVIG during pregnancy and breastfeeding. Therapeutic plasma exchange could be beneficial as an alternative in the first trimester of pregnancy.

## Breastfeeding

The PRIMS study found no difference in ARR in the first 3 months postpartum between women who did not breastfeed (ARR 1.3) and those who did for any duration (ARR 1.2)^50^. But other studies – including a meta-analysis – indicate that breastfeeding, independent of its duration, might actually have a protective effect on the postpartum relapse rate and delay the timing of a relapse^89–91^. The relationship between disease activity and breastfeeding has been clearly demonstrated and shows that the occurrence of a postpartum relapse relates only to preconception and antenatal disease activity, but not to breastfeeding itself^91^. In the first month after delivery the patient’s clinical condition and neuroimaging status should be reviewed.

Some studies reported that exclusive breastfeeding (less than one bottle a day) for at least 2 months post-delivery may reduce 6 months postpartum relapse risk^92^ while others showed that breastfeeding had little or no influence on postpartum relapses^93^. This raises the question whether the protective effect of breastfeeding on MS relapses was related to the confound of selection bias in the choice to breastfeed, in that patients with milder MS choose to breastfeed as compared with their counterparts with more active disease.

Very few studies provide information about DMTs and their excretion in breast milk and their potential effects on the newborn^94^. The use of these DMTs will be discussed in the DMD-related sections.

Although the potential increased risk of relapses postpartum would favor immediate restart of DMTs, the optimal time of resuming treatment after delivery has not been defined yet. Moreover, conclusive data that the early reintroduction of DMTs reduces the postpartum relapse risk are still lacking^95^.

The decision to resume or start a DMT immediately after birth, particularly in women at higher risk of increased disease activity, needs to be weighed against the potential benefits of breastfeeding in the setting of a shared decision-making process. However, in women with high preconception disease activity and those who do not wish to breastfeed, treatment should not be postponed and early (during the first 10 days postpartum) reintroduction of DMTs may be recommended^96^. In support of this, early administration of IFNβ or GA (within 3 months postpartum) have proven to reduce the risk of reactivation by 50%^97^ and significantly reduce the risk of relapses postpartum and over a follow-up period of at least 1 year^95^. Similarly, natalizumab started within 8 days of delivery prevented postpartum relapses in five of six highly active MS patients^79^.

*Recommendations*: Women with MS should be encouraged to breastfeed with a possible favorable effect of exclusive breastfeeding. To avoid risk of relapses postpartum, early reintroduction of DMTs may be advised.

## The use of DMTs before, during and after pregnancy

Women with MS should be informed that none of the currently licensed DMDs interact with hormonal contraception^80^.

Discussions on the benefit/risk profile of DMTs before, during and after pregnancy should occur ideally at or soon after the diagnosis, and should be repeated regularly thereafter. This would avoid stressful situations and exposure to potentially teratogenic drugs^79^. Treatment of MS during pregnancy should be tailored to the patient’s needs, taking into consideration: age, previous relapses and their severity, MRI activity, disease progression, disability, the risk of treatment discontinuation vs. maintaining therapy, and last but not least the patient’s preference.

Because of the small number of pregnancies that occurred during the clinical trials, premarketing data precludes recommendations regarding safety of DMDs during pregnancy. Therefore most DMTs are generally not recommended for use during pregnancy unless the benefits outweigh the potential risks to the fetus. If required, washout period for the DMDs should be as short as possible. However women with highly active MS requiring washout of any DMD are exposed to increased risk of relapse and may benefit of monthly pulse doses of corticosteroids until pregnancy is confirmed^32^.

As the risk of relapses in the postpartum period is independently correlated with the 12-month and 24-month ARR preceding pregnancy^52^^,^^53^^,^^95^, it is essential that disease activity is stable for at least one year prior to conception^37^.

The use of most DMDs are contraindicated during lactation and the decision to resume DMDs shortly after delivery should involve careful consideration of potential risk of rebound of disease activity in the postpartum period and adverse effects on the infant versus benefits from breastfeeding.

The mechanism of action and adverse effect profile of the different DMTs have been the subject of previous publications^98^^,^^99^. In this section, the DMTs have been classified as injectable, oral and infusion DMDs. Their relevant pharmacokinetic properties and use during pregnancy and breastfeeding have been summarized in Table 1.

**Table 1.** Approved disease modifying therapies (DMTs) for MS: overview of their use before, during and after pregnancy.

DMT	Relevant pharmacokinetic properties	Wash-out period preconception	Use in pregnancy (P) and during breastfeeding (BF)	Effect in neonate	Practical recommendations with regard to pregnancy and breastfeeding
Injectable DMDs					
Interferon beta (IFNβ)	MW ≈ 18.5–22.5 kDa t½ ≈ 10–69h not crossing placenta	Not required	P: No increased risk of fetal abnormalities or adverse pregnancy outcomes (*N* > 3,500) BF: Theoretically safe (RID <0.006%)		Pregnancy: Stop contraception. Continue IFNβ at least until pregnancy confirmed. Continue through entire pregnancy depending disease activity. In unplanned pregnancy, continue IFNβ depending disease activity. Breastfeeding: Considered safe. (Benefit of breastfeeding outweighs the risk of treatment).
Glatiramer acetate (GA)	MW ≈ 5–9 kDa t½ ≈ 20h not crossing placenta	Not required	P: No increased risk of fetal abnormalities or adverse pregnancy outcomes (*N* > 8,000) BF: Theoretically safe		Pregnancy: Stop contraception. Continue GA at least until pregnancy confirmed. Continue through entire pregnancy depending disease activity. In unplanned pregnancy, continue GA depending disease activity. Breastfeeding: Considered safe. (Benefit of breastfeeding outweighs the risk of treatment).
Oral DMDs					
Teriflunomide	MW ≈ 270Da t½ 15–18 days high PB extensive enterohepatic recycling crosses placenta	Use accelerated elimination procedure	P: No evidence of increased risk of fetal abnormalities or adverse pregnancy outcomes (*N* > 500) BF: Contraindicated: likely transfer but no data		Pregnancy: Discontinue teriflunomide before conception Use accelerated elimination procedure. Maintain effective contraception as long as teriflunomide plasma concentration is > 0.02 mcg/mL. Switch to an alternative treatment if required. In unplanned pregnancy accelerated elimination procedure should be done as soon as possible. Breastfeeding: Contraindicated during treatment.
Dimethyl fumarate (DMF)	MW ≈ 144Da t½ ≈ 1h high Vd crosses placenta	Not required	P: No evidence of increased risk of fetal abnormalities or adverse pregnancy outcomes (*N* < 200) BF: Contraindicated: likely transfer but no data		Pregnancy: Discontinue DMF before conception. If required start alternative treatments e.g. natalizumab. In unplanned pregnancy discontinue DMF immediately. Breastfeeding: Contraindicated during treatment.
Fingolimod	MW ≈ 307Da high oral AUC t½ 6–9 days high PB crosses placenta	2 months	P: No evidence of increased risk of fetal abnormalities or adverse pregnancy outcomes (*N* < 500) BF: Contraindicated: transferred		Pregnancy: Discontinue fingolimod before conception. Maintain effective contraception for at least 2 months. Consider alternative treatments e.g. natalizumab. In unplanned pregnancy discontinue fingolimod immediately. Breastfeeding: Contraindicated during treatment.
Cladribine	MW ≈ 285Da t½ 6–20h crosses placenta	6 months after the last course	P: No evidence of increased risk of fetal abnormalities or adverse pregnancy outcomes (*N* < 50) BF: Contraindicated: likely transfer but no data		Pregnancy: Maintain effective contraception for at least 6 months after last course. Discontinue any further treatment until after pregnancy. Breastfeeding: Contraindicated during treatment and for 1 week after the last dose.
Siponimod	MW ≈ 516Da t½ ≈ 57h cytochrome P450 2C9 dependent crosses placenta	Not available	P: Limited data BF: Contraindicated: likely transfer but limited data		Pregnancy: Discontinue siponimod before conception. Maintain effective contraception for at least 10 days. Discuss alternative treatments. In unplanned pregnancy discontinue siponimod immediately. Breastfeeding: Contraindicated during treatment
Infusion DMDs					
Natalizumab	MW ≈ 150 kDa t½ ≈ 11 days ± 4days Prolonged t_ss_ 28 weeks crossing placenta in 2nd and 3rd trimester	Not required	No evidence of increased risk of fetal abnormalities or adverse pregnancy outcomes (*N* > 500) BF: Contraindicated: transferred in human breast milk (RID 2–5% at 50 days)	After week 30 reversible hematological abnormalities in newborn	Pregnancy: Re-active approach: Discontinue natalizumab before conception. Continue effective contraception for at least 2 months after discontinuation. If required, resume in case of relapse. Pro-active approach: Stop contraception. Continue natalizumab at least until pregnancy confirmed. Continue natalizumab after conception. Use EID of natalizumab during pregnancy. Last dose during pregnancy at latest week 34 and resume soon after birth. In unplanned pregnancy continue natalizumab in highly active disease. Breastfeeding: Contraindicated during treatment but if women decide to breastfeed under natalizumab, infants should be monitored for hematological abnormalities.
Alemtuzumab	MW ≈ 150 kDa t½ 4–5 days crosses placenta	4 months after the last course	P: No evidence of increased risk of fetal abnormalities or adverse pregnancy outcomes (*N* < 200) BF: Contraindicated: unlikely transfer but no data	Potential for transient neonatal Graves’ disease	Pregnancy: Maintain effective contraception for at least 4 months after last infusion. Discontinue any further treatment until after pregnancy. Breastfeeding: Contraindicated during treatment.
Ocrelizumab	MW ≈ 145 kDa t½ ≈ 26 days crosses placenta	FDA: 6 months after the last infusion EMA: 12 months after the last infusion	P: No evidence of increased risk of fetal abnormalities or adverse pregnancy outcomes (*N* < 50) BF: Contraindicated: unlikely transfer but no data	Reversible reduction in B-cell count in newborn Plan vaccinations accordingly	Pregnancy: Maintain effective contraception for at least 6 months after the last infusion. Discontinue any further treatment until after pregnancy. Breastfeeding: Contraindicated during treatment.

Abbreviations. AUC, area under the curve; DMD, disease modifying drug; EID, extended interval dose; EMA, European Medicines Agency; FDA, Food and Drug Administration; MW, molecular weight; PB, protein binding; RID, relative infant dose (compared with maternal dose); t½, terminal half-life; t_ss_, time to steady state; Vd, volume of distribution.

*Recommendations*: None of the currently licensed DMDs interact with hormonal contraception. Treatment and usage of DMDs during pregnancy should be tailored toward the patient’s needs. The use of most DMDs is contraindicated during breastfeeding.

## Injectables: interferon beta and glatiramer acetate

### Interferon beta (IFNβ)

IFNβ is a polypeptide with a molecular weight (MW) – depending the type of IFNβ – varying between 18.5 kDA (IFNβ-1b) and 22.5 kDa (IFNβ-1a)^100^, and hence does not cross the placenta^101^. IFNβ is classified as pregnancy category C (FDA)/category 2 (EMA).

Registry-based cohorts and worldwide databases have compiled data of over 3,500 pregnancies exposed to IFNβ. Most of these exposures occurred in the early weeks of the first trimester followed by discontinuation of IFNβ once pregnancy was confirmed. All studies revealed no increased risk of either spontaneous abortions or congenital anomalies compared with the general population^59^^,^^93^^,^^102–104^. A systematic review of perinatal and developmental outcomes in offspring of women exposed to IFNβ *in utero* (761 pregnancies) showed lower mean birth weight, lower mean gestational age, and an increased incidence of preterm birth^105^.

Few data are available for IFNβ exposure during the second and third trimester of pregnancy. One study (seven pregnancies) reports on IFNβ treatment beyond the first trimester without adverse pregnancy outcomes^102^.

Recently the EMA has updated the label of IFNβ allowing, if clinically needed, the use during pregnancy and lactation^106^. Hence in case of unplanned pregnancy treatment continuation with IFNβ can be considered if the benefit to the mother outweighs the potential risk to the fetus. Although continuing treatment with IFNβ throughout pregnancy may not be required for some women the decision to continue should be based on the level of disease activity prior to the pregnancy. There is no evidence that treatment continuation with IFNβ will result in harm to the fetus^59^^,^^93^^,^^102–104^. The benefits of treatment continuation include a potential reduction in the frequency and severity of postpartum relapses, although it is impossible to predict in which patients relapses will occur. The pros and cons of treatment continuation throughout pregnancy should be discussed with the patient providing her with a chance to make an informed choice.

With regard to breastfeeding, due to its high MW only small amounts (0.006% of maternal dose) of IFNβ are excreted in breast milk^107^. In addition, when given orally, IFNβ has no systemic biological effect^108^. Therefore, women who intend to breastfeed may use IFNβ without concerns that this might affect the newborn.

There is no obstetric or neonatal risks for the offspring of men undergoing treatment with IFNβ who father children^9^.

*Recommendations*: Treatment with IFNβ until pregnancy is confirmed is safe^109^. If clinically needed IFNβ can be used during the entire pregnancy. Breastfeeding under IFNβ is considered safe.

### Glatiramer acetate

Glatiramer acetate (GA) is a large polypeptide with a MW between 5–9 kDa and because of its large size does not cross the placenta^110^.

GA is the only DMD with a pregnancy category rating B (FDA)/category 2 (EMA).

Clinical data from several large studies and pharmacovigilance databases involving more than 8,000 pregnancies exposed to GA – most of them with first trimester exposure – did not reveal any adverse pregnancy outcomes^54^^,^^111–114^. Hence GA is no longer contraindicated in pregnancy and can be used if the benefit to the mother outweighs the risk to the fetus.

Few data are available on GA exposure during the entire pregnancy, none of them showed increased risk of adverse pregnancy outcome^54^^,^^111^^,^^114^^,^^115^.

No data are available on the excretion of GA in breast milk. But provided the large MW of GA, crossing into breast milk is very unlikely and hence nursing is potentially safe. There appears to be no adverse effect of maternal GA on breastfed babies^116^.

No association between paternal exposure to GA at the time of conception and a risk of adverse outcomes has been shown^9^^,^^117^.

*Recommendations*: If clinically required GA can be continued during the entire pregnancy. Breastfeeding under GA is probably safe.

## Oral DMDs: teriflunomide, dimethyl fumarate, fingolimod, cladribine and siponimod

### Teriflunomide

Teriflunomide is a small molecule with a MW of 270 Da. Its mean plasma half-life is 15–18 days. However following discontinuation of the drug, it may take 8 months up to 2 years to reach a negligible serum concentration of <0.02 mcg/mL^118^. This is due to its extensive enterohepatic recycling. Therefore after treatment discontinuation, an accelerated washout procedure with oral cholestyramine (8 gram three times per day for 11 days) or activated charcoal is recommended^119^. Teriflunomide is classified as category X (FDA)/category 1 (EMA).

Despite all the concerns regarding teratogenicity in animal studies, pregnancy registries from clinical trials and post-marketing surveillance reports – the largest one containing 437 confirmed teriflunomide-exposed pregnancies^120^ – have not revealed any teratogenic signals^119–121^.

Despite this, women of childbearing potential are still advised to use effective contraception while treated with teriflunomide and for 2 years after discontinuation of the drug unless accelerated elimination procedure has been used. Pregnancy planning while on teriflunomide requires a careful and timely strategy. Teriflunomide treatment should be discontinued if conception is desired or an unintended pregnancy arises, and an accelerated elimination procedure started followed by measuring of the serum level of the drug (target level <0.02 mcg/mL)^122^. Switching to an alternative DMD e.g. IFNβ, GA or natalizumab should be considered depending on the level of disease activity.

There are no data on teriflunomide exposure during second and third trimester of pregnancy.

Pregnancies while on teriflunomide are considered high risk and fetal organ screening by means of ultrasound is recommended.

Being a small molecule, teriflunomide is likely excreted into breast milk and is therefore contraindicated during lactation^122^.

Teriflunomide can reduce sperm count^119^. With regard to fathering offspring, the FDA label of teriflunomide states that man wishing to father a child should discontinue teriflunomide therapy and undergo accelerated wash-out, while the EMA considers the risk of male-mediated embryofetal toxicity through teriflunomide therapy low^123^^,^^124^.

*Recommendations*: Teriflunomide is not recommended during pregnancy and breastfeeding. In case of desired conception or unplanned pregnancy when treated with teriflunomide, the use of accelerated elimination procedure (cholestyramine or activated charcoal) is highly recommended.

### Dimethyl fumarate

Dimethyl fumarate (DMF) is a small molecule with a MW of 144 Da and terminal half-life of 1 h^125^. Therefore, pregnancy planning following withdrawal from DMF should not pose a real problem. DMF received a pregnancy category C (FDA)/category 2 (EMA).

In animal models, the drug has shown to cross the placenta but no malformations were observed at nontoxic doses^126^. At very high doses and toxic doses low birth weight, delayed ossification, and a higher risk for spontaneous abortion were observed^126^.

However, in clinical trials and post-marketing surveillance studies, no increased risk of fetal abnormalities or adverse pregnancy outcomes have been observed compared with the general population^126–128^.

There are no data of pregnancy outcomes while treated with DMF beyond the first trimester of pregnancy.

Women of childbearing potential are advised to use effective contraception while treated with DMF. Because of the short half-life no washout period is required. Switching to an alternative DMD e.g. IFNβ, GA or natalizumab should be considered depending on the level of disease activity. However as DMF treatment may be associated with a significant drop in absolute lymphocyte, CD4, and CD8 counts, treatment with natalizumab should not be initiated before meaningful lymphocyte reconstitution (≥800/µL) occurs, which generally takes places 6–8 weeks after DMF discontinuation^98^.

Thus far there are no reports of acute disease reactivation following discontinuation of DMF.

There are no data with regard to excretion of DMF or its metabolite in breast milk, but the low MW of the drug and its metabolite make this plausible. Therefore, administration during lactation should be avoided.

No recommendation has been provided by drug agencies regarding paternal exposure to DMF at the time of conception and risk of adverse outcomes.

*Recommendations*: DMF is not recommended during pregnancy and lactation. No wash-out period is required, but lymphocyte re-population is required before switching to natalizumab.

### Fingolimod

Fingolimod is an oral sphingosine-1-phosphate (S1P) agonist with a MW of 307 Da which is able to cross the placenta^7^^,^^129^. In the embryo, the S1P receptor is involved in the organogenesis of blood vessels and the heart. Not surprisingly, in animal studies, fingolimod has been associated with embryolethal and teratogenic effects^7^^,^^129^ such as ventricular septal defect and persistent truncus arteriosus. These teratogenic effects occurred at doses lower than those recommended in humans. Although fingolimod may affect liver function it does not seem to interfere with pharmacokinetics of oral contraceptives or their contraceptive efficacy^130^.

Data from the clinical development program^131^ revealed 5 cases (7.6%) of abnormal fetal development in the 66 pregnancies that had *in utero* exposure to fingolimod. In all five cases, fetal exposure to the drug happened in the first trimester of pregnancy. More recently, results of a fingolimod-exposure registry (1,246 pregnancies) that included three prospective database sources showed that the prevalence of major congenital malformations or miscarriages was not higher among pregnant women exposed to fingolimod compared with the general population and the unexposed MS population^132^. In particular, the prevalence of cardiac malformations observed was not significantly different from that of the general population. In addition, the proportion of miscarriage was in line with those of the general and unexposed MS population and no specific pattern of birth defects was identified. Regardless of this data, fingolimod is pregnancy category C (FDA)/category 2 (EMA) and hence should be avoided during pregnancy. This was also confirmed after review of post-marketing data and several registries that fingolimod exposure in pregnancy carries a two-fold increased risk of congenital malformations (congenital heart diseases (such as atrial and ventricular septal defects, tetralogy of Fallot), renal abnormalities and musculoskeletal abnormalities) compared with the observed rate of 2–3% in the general population. Consequently the EMA updated the label of fingolimod^133^. It is therefore recommended to discontinue fingolimod at least 2 months prior to conception while using effective contraceptive measures and closely monitor the patient^131^^,^^132^^,^^134^. In case of unplanned pregnancy, fingolimod should be discontinued immediately and as these are high risk pregnancies, fetal organ screening ultrasound is recommended.

Pregnancy does not protect against relapses following the cessation of fingolimod, and some women experience severe rebound relapses after withdrawal of the drug during pregnancy^135^^,^^136^. To reduce this risk of relapse during withdrawal, patients could be switched to natalizumab prior to pregnancy. However this treatment should not be initiated before meaningful lymphocyte reconstitution (≥800/µL) has occurred, which generally takes places 6–8 weeks after fingolimod cessation^7^^,^^98^.

Fingolimod can be identified in human breast milk, and consequently the treatment should not be resumed if the mother intends to breastfeed^80^.

There are no data on reduced paternal fertility in men exposed to fingolimod.

*Recommendations*: Fingolimod should be avoided during pregnancy and lactation. Effective contraception is recommended during fingolimod therapy and a 2-month wash-out period before conceiving. In women with highly active disease switch to natalizumab may be considered prior to conception, once lymphocyte reconstitution has occurred.

### Cladribine

Claribine is a purine nucleoside analog which has recently been approved by FDA for RRMS and active secondary progressive MS and by EMA for highly active relapsing MS^137^^,^^138^. The drug is an example of oral selective pulse immune reconstitution therapy. It is a small molecule with a MW of 285 Da which crosses the placenta. Since its mode of action is based on toxic impact of its main metabolite, 2-chlordeoxyadenosine triphosphate (2-CdATP) on cells resulting in apoptosis, it may carry adverse effects on gametogenesis and embryogenesis.

Based on this, cladribine is classified as category D (FDA)/category 2 (EMA).

There are no data of cladribine-exposed pregnancies or breastfeeding in MS, but there are a few case reports with good outcomes in cladribine-exposed pregnancies in the context of its use in hairy cell leukemia^139^^,^^140^.

It is strongly recommended to avoid conceiving during cladribine treatment and respect at least a 6-month period after each course of the drug before considering conception. In addition, both a hormonal and barrier contraceptive method should be considered for at least 4 weeks after the last dose in each treatment year^141^.

In view of its mechanism of action, adverse effects on gametogenesis are very likely. Therefore fathering while on cladribine should be delayed until at least 6 months after the last dose.

*Recommendations*: Women receiving cladribine should avoid pregnancy and lactation. Effective contraception is recommended during treatment and for at least 6 months after the last dose. Similarly, fathering while on cladribine should be delayed until at least 6 months after the last dose.

### Siponimod

Siponimod is a selective sphingosine-1-phosphate (S1P) receptor modulator for the S1P1 and S1P5 receptor. The drug was recently approved by the FDA for the treatment of adults with RRMS, active secondary progressive MS, and clinically isolated syndromes^142^. The drug has a MW of 516 DA and a half-life of approximately 30 h, and is contraindicated in patients with CYP2C9*3/*3 genotype^143^. Placental transfer of siponimod and metabolites has been shown in animal studies.

The drug reveals a reproductive toxicity in animal studies comparable with that of fingolimod^142^.

To date there are no clinical data available on the use of siponimod-exposed pregnancy and breastfeeding.

Women of childbearing potential should use effective contraception during and for 10 days after stopping the drug due to the potential risk of fetal harm^144^.

*Recommendations*: Women receiving siponimod should avoid pregnancy and lactation. Effective contraception is recommended during treatment with siponimod.

## Infusion DMDs: natalizumab, alemtuzumab and ocrelizumab

### Natalizumab

Natalizumab is an IgG4 humanized monoclonal antibody which binds to the α4-integrin receptor of the lymphocytes. Natalizumab is a very large molecule with a MW 150 kDa, making its crossing in the placenta during the initial stages of pregnancy virtually impossible^8^. However, from the second trimester onwards the drug is actively transported over the placenta leading to increasing fetal serum levels when exposure occurs.

Animal studies revealed that natalizumab exposure did not result in teratogenicity but was associated with abortifacient effects^145^^,^^146^. An exposure throughout pregnancy revealed hematological abnormalities in the offspring of primates, which were reversible following natalizumab elimination^146^.

The pregnancy exposure registry of natalizumab revealed that spontaneous abortion occurred in 9% of pregnancies and major congenital abnormalities in 5% of the 363 natalizumab-exposed pregnancies^147^. Overall, 8.3% major or minor birth defects were reported, with no specific patterns of malformations that would suggest a drug effect. Data from a German MS pregnancy registry confirmed that there is no increased risk for congenital abnormalities after natalizumab exposure during the first trimester of pregnancy compared with healthy controls^148^ or diseased controls without natalizumab exposure^149^. These findings were also confirmed in an Italian population of 92 natalizumab-exposed pregnancies^150^. However compared with IFNβ-exposed pregnancies there was a 13% higher spontaneous abortion rate in the natalizumab-exposed pregnancies. Although there is still some controversy, the EMA does not rate the malformation risk for natalizumab higher nor confirms a certain pattern of malformations.

Given it teratogenic effects in animal models natalizumab was granted a category C (FDA)/category 2 (EMA) and should be used during pregnancy only if the potential benefit to the patient justifies the potential risk to the fetus.

Natalizumab withdrawal before or during pregnancy may result in severe disease reactivation or even disease rebound which usually occurs 12–16 weeks after discontinuing the drug and which may lead to severe disability^151–154^. In fact, disease reactivation (clinically and/or neuroradiologically) has been reported in 95.5% of patients with MS who discontinued natalizumab due to pregnancy^155^. In addition, almost one third of these patients experienced worsening of disease disability. Hence, natalizumab withdrawal during pregnancy might not be an option and the potential risks and benefits of discontinuing versus maintaining natalizumab should be thoroughly discussed with the patient at the onset of the treatment and before conception.

In an attempt to prevent breakthrough disease activity, extended interval dosing (every 6–8 weeks instead of standard 4 weeks interval) has proven effective and may be advised throughout the pregnancy to reduce exposure to natalizumab^156^^,^^157^. The drug should then be discontinued in the last trimester (at the latest by week 32–34) and restarted shortly after delivery. Reversible hematological abnormalities (thrombocytopenia and hemolytic anemia) have been observed in over 75% of newborn at delivery from mothers treated with natalizumab throughout pregnancy with only few newborns requiring blood transfusions^158^. Generally, these hematological abnormalities return to normal within 4 months postpartum, after exposure during the third trimester^159^.

Few case reports show that natalizumab exposure in the third trimester of pregnancy results in normal pregnancy outcomes^160–162^. In these reports, all hematological changes observed in the neonate were reversible.

Routine MRI monitoring for progressive multifocal leukoencephalopathy in John Cunningham virus positive women should be continued during pregnancy.

Natalizumab has been detected in the serum of the newborn and in breast milk samples. Cell-bound natalizumab was measurable in both mother-baby pairs with significant higher levels in babies^163^^,^^164^. If women decide to breastfeed under natalizumab, infants should be monitored for hematological abnormalities.

There is no evidence that natalizumab reduces fertility in man.

*Recommendations*: The following approaches can be considered: the most conservative approach is to discontinue natalizumab before conception and maintain effective contraception for 2–3 additional months after discontinuation of the drug. However since most woman treated with natalizumab are likely to have high disease activity and considering the potential risk of severe relapses or even rebound disease activity after withdrawal, it seems reasonable to continue natalizumab throughout the pregnancy (active approach) with extended infusion intervals of 6–8 weeks and discontinue the drug in the last trimester (week 32–34). The newborn should be screened for potential hematological abnormalities. Infants should be monitored for hematological abnormalities during breastfeeding.

### Alemtuzumab

Alemtuzumab is a recombinant humanized monoclonal antibody that binds to the CD52 receptor, expressed on the surface of T and B-lymphocytes and natural killer cells. The drug has a MW of 150 kDa. It has a relatively short half-life of 4–5 days and is completely eliminated after 30 days^165^. Alemtuzumab is listed as pregnancy category C (FDA)/category 1 (EMA).

Animal studies have shown increased potential for spontaneous abortion and reduced fetal lymphocyte count while exposed to the drug *in utero*^165^.

As for most monoclonal antibodies, alemtuzumab does not cross the placental barrier in the first trimester of pregnancy. Therefore, at least in theory, conception occurring while alemtuzumab is being used should raise no particular concerns regarding teratogenicity, and no particular recommendations for washout should be necessary. However after week 20 the fetus will be subjected to the effects of the monoclonal antibody.

Data from the clinical development program of 248 pregnancies in alemtuzumab-treated women did not reveal any congenital abnormalities or birth defects and the rate of spontaneous abortion was comparable with that observed in treatment-naïve MS patients and general populations^166^.

Despite this, women of childbearing potential should use effective contraceptive measures during treatment with alemtuzumb and for at least 4 months after the last infusion (as most of the pregnancies occurred at least 4 months after last infusion in the clinical development program of the drug). It is mandatory to perform a pregnancy test prior to initiation of each alemtuzumab course. The potential for secondary autoimmune diseases e.g. autoimmune thyroid diseases might favor the risk for miscarriages, intrauterine growth retardation, preeclampsia and preterm birth (hyperthyroidism) or irregular menstruation, infertility, and a restricted mental development of the child (hypothyroidism). In addition, maternal thyroid-stimulating hormone receptor antibodies can cross the placenta and cause transient neonatal Graves’ disease^165^. Consequently, in case of known autoimmune thyroid disease monitoring of thyroid hormones should be done monthly during pregnancy. In case of maternal thyroid disease, the infant should be tested as well.

There are limited data beyond the first trimester of pregnancy.

As with most monoclonal antibodies, alemtuzumab is excreted in breast milk and degraded and as of now breastfeeding is not recommended^165^.

There are no adequate clinical safety data on the effect of alemtuzumab on fertility in male.

*Recommendations*: Women receiving alemtuzumab should avoid pregnancy. Effective contraception is recommended during treatment and up to 4 months after each course of treatment. Lactation is not recommended for at least 4 months after the last infusion of each treatment course.

### Ocrelizumab

Ocrelizumab is a recombinant humanized monoclonal antibody directed against CD20 expressed on most B-lymphocytes. The drug has been approved in active relapsing and primary progressive forms of MS^167^^,^^168^. Ocrelizumab has a MW of approximately 145 kDa and is able to cross the placenta after the first trimester^168^. Its half-life is 26 days^169^.

In primates, administration of ocrelizumab during fetal organogenesis did not result in embryocytotoxic or teratogenic effects and had no effect on abortion or embryo-fetal fatality rate. However, ocrelizumab was associated with peripheral B-cell depletion and immunosuppression in both the mother and offspring. B-cell reconstitution occurred by 6 months of age^170^.

Data from 267 pregnancies in ocrelizumab-treated women do not suggest an increased risk of adverse outcomes^170^. Nevertheless, women of childbearing potential should use effective contraception while receiving the drug for at least 6 months (FDA recommendation) up to 12 months (EMA recommendation) after the last infusion of ocrelizumab^171^^,^^172^. If ocrelizumab infusions were to be used during pregnancy, the neonate’s B-cell count should be checked in the umbilical cord blood of the newborn and vaccination should be planned accordingly. Newborns without B-cells should not receive vaccination and this until B-cell reconstitution has occurred (usually after 6–10 months).

Low concentrations of ocrelizumab were detectable in breast milk, but despite this breastfeeding is not recommended^167^^,^^168^.

There are no adequate clinical safety data on the effect of ocrelizumab on fertility in male.

*Recommendations*: Ocrelizumab should be avoided during pregnancy unless the potential benefit to the mother outweighs the potential risk to the fetus. Women should be advised to delay conception at least 6 month from last infusion. Lactation is not recommended for at least 6 months after last infusion. B-cells should be monitored in the newborn and vaccination delayed till B-cell reconstitution has occurred.

## The use of symptomatic treatment during and after pregnancy

In addition to their MS symptoms, pregnant and lactating women with MS may face other conditions such as urinary tract infections, incontinence, depression, fatigue, spasticity or gait abnormalities, which can all worsen during and after pregnancy. The management of these conditions includes the use of oral drugs many of them with small MW and almost all of them are categorized FDA pregnancy risk B, C and D^173^. Except for a few antidepressants the safety data of these drugs is very scanty. Clinical data on the use of antidepressants by pregnant women and nursing mothers revealed that these drugs are essentially not teratogenic^174–175^. Sertraline has been well-studied during pregnancy with more than 10,000 sertraline-exposed pregnancies during the first trimester. The drug shows little interaction and has a linear pharmacokinetic profile with a half-life of 24–26 h. The drug may also be beneficial for treating depression during breastfeeding^176^.

Drugs used in the management of fatigue (amantadine, modafenil) (FDA pregnancy category C) and urinary incontinence due to overactive bladder e.g. oxybutynin (FDA pregnancy category B) are not recommended during pregnancy and lactation, and should be used only if the potential benefit to the patient outweighs the risk to the patient and fetus.

Limited data are available on fampridine which is recommended for improving gait velocity in MS. The drug is hence not recommended for women who intend to become pregnant or breastfeed. Similarly, tizanidine and baclofen used for the management of spasticity should not be prescribed at any time during preconception or pregnancy. There is however no contraindication for breastfeeding while using either oral or intrathecal baclofen, as the drug is detected only in small amounts in breast milk^177^^,^^178^.

The use of natalizumab during pregnancy may cause reactivation of herpes infection. Fortunately, there is accumulating evidence from large pregnancy registries that oral acyclovir and valacyclovir are safe during pregnancy^179^^,^^180^.

A recently published German retrospective study revealed that less than 2% of patients suffering from MS have concomitant seizures or epilepsy, 77% of them being focal in nature^181^. There are no specific guidelines to the use of anti-epileptic drugs in MS particularly during pregnancy, but general recommendations advise drugs like carbamazepine, oxcarbazepine, lamotrigine or levetiracetam as first choice^182^.

With regard to infections in pregnant woman with MS – most commonly urinary tract infections (UTI) – oral nitrofurantoin is considered the first choice in asymptomatic bacteriuria and acute cystitis, followed by amoxicillin and cephalexin. Parenteral antibiotic therapy may be required in women with pyelonephritis. The latter should be guided by urine culture and sensitivity reports as soon as available^183^. *Recommendations*: Discontinue any symptomatic drug before conception or use the lowest effective dose for the shortest time possible while balancing risk/benefit. Counseling patients before pregnancy and an in-depth discussion with patient and caregivers about any possible need and selection of any symptomatic drug is paramount. Acyclovir and valacyclovir are considered safe during pregnancy.

## Conclusion

MS has no effect on fertility, pregnancy and fetal outcomes, and pregnancies do not have a negative impact on the long-term disease course and accumulation of disability. There is a potential risk of having a relapse after use of gonadotropin-releasing hormone agonists during ART. In the short term, pregnancy leads to a reduction of relapses during the third trimester, followed by a short-term increased risk of relapse during the first three months postpartum. Pregnancies in women with MS are not per se high risk pregnancies and MS does not influence the mode of delivery or anesthesia unless significant disability is present. MRI is not contraindicated at any time during pregnancy but should only be considered if it is absolutely indicated and the findings could have therapeutic consequences. MRI with gadolinium contrast media should be avoided where possible. For the management of acute disabling relapses during pregnancy and breastfeeding, pulse dose methylprednisolone infusion can be used. However their use during the first trimester of pregnancy is still controversial and should preferably be avoided. Women with MS should be encouraged to breastfeed with a possible favorable effect of exclusive breastfeeding.

In clinical practice, DMDs can be classified according to their potential pregnancy-associated risk and fetal outcome; the first category refers to those DMDs which can be continued until pregnancy is confirmed and include GA, IFNβ, and natalizumab. Once pregnancy is confirmed a decision needs to be taken on, treatment continuation/discontinuation taking into consideration the benefit to the mother and potential risk to the fetus. The second category refers to those DMDs that should be discontinued prior attempting to conceive, and for which effective contraception during the washout period is highly recommended. These DMDs include teriflunomide, DMF, fingolimod, cladribine, siponimod, alemtuzumab, and ocrelizumab. In case of unintended pregnancy, these DMDs should be immediately discontinued. Switching to a safer DMD (GA, IFNβ, and natalizumab) and if clinically needed to be continued during the pregnancy, is an option depending on the individual patient.

In women who are perceived to be at high risk of relapse or those with highly active MS, the continuation of natalizumab during pregnancy is probably the best option currently available.

Exclusive breastfeeding for the first 2 months postpartum may be independently associated with decreased postpartum relapse rate.

The second category of DMDs should be avoided during breastfeeding. In case of high disease activity and those women who prefer not to breastfeed, the early (7–10 days postpartum) reintroduction of DMT should be considered.

Decision-making in a woman with MS is a shared process between patient and physician, and the approach with regard to most pre-, peri- and postpartum issues must be individualized for each patient.

## Transparency

### Declaration of financial/other relationships

BC and DD have presented at meetings and served on Novartis, Sanofi, Roche and Merck advisory boards. The other authors have no financial disclosure to declare. The peer reviewers on this manuscript have no relevant financial or other relationships to disclose.
